# Association of the host genetic factors, hypercholesterolemia and diabetes with mild influenza in an Iranian population

**DOI:** 10.1186/s12985-021-01486-3

**Published:** 2021-03-25

**Authors:** Parvaneh Mehrbod, Sana Eybpoosh, Behrokh Farahmand, Fatemeh Fotouhi, Majid Khanzadeh Alishahi

**Affiliations:** 1 Influenza and Respiratory Viruses Department, Pasteur Institute of Iran, Tehran, Iran; 2 Department of Epidemiology and Biostatistics, Research Centre for Emerging and Reemerging Infectious Diseases, Pasteur Institute of Iran, Tehran, Iran; 3 Private Practitioner, Mashhad, Iran

**Keywords:** Influenza, Genetic polymorphisms, Hypercholesterolemia, Diabetes, Iran

## Abstract

**Background:**

Variation in host genetic factors may result in variation in the host immune response to the infection. Some chronic diseases may also affect individuals’ susceptibility to infectious diseases. The aim of this study was to evaluate the association of the host genetic factors mostly involved in inflammation, as well as hypercholesterolemia and diabetes with mild flu in an Iranian population.

**Methods:**

In this cross-sectional study, nasopharyngeal swab samples were collected from 93 patients referred to primary care centers of Markazi, Semnan, and Zanjan provinces (central Iran) due to flu-like symptoms between March 2015 and December 2018. Of these, PCR test identified 49 influenza A/H1N1 and 44 flu-negative individuals. Twelve single-nucleotide polymorphisms (SNPs) in RPAIN, FCGR2A, MBL-2, CD55, C1QBP, IL-10, TNF-α and an unknown gene were genotyped using iPLEX GOLD SNP genotyping analysis. Hypercholesterolemia and diabetes status was determined based on the physician diagnosis. Association of the host genetic variants, hypercholesterolemia and diabetes with mild A/H1N1 flu was assessed with univariable and multivariable logistic regression analysis as implemented in Stata software (v.14). Statistical tests were considered as significant at 0.05 levels.

**Results:**

Frequency of diabetes and hypercholesterolemia, as well as participants mean age was significantly higher in the flu-negative rather than the flu-positive group. Of 12 SNPs, nine did not show any significant association with mild flu in our study (rs1801274, rs1800451, rs2564978, rs361525, rs1800450, rs1800871, rs1800872, rs1800896, rs1800629). Possessing G vs. A allele in two SNPs (rs3786054 and rs8070740) was associated with a threefold increase in the chance of mild flu when compared to flu-negative patients (95% CI: 1.1, 22.0). Possessing C allele (vs. A) in the rs9856661 locus also increased the chance of mild flu up to 2 folds (95% CI: 1.0, 10.0).

**Conclusion:**

The results showed that possessing the G allele in either rs3786054 or rs8070740 loci in C1QBP and RPAIN genes, respectively, increased the risk of H1N1 infection up to 3.3 folds, regardless of the patient’s age, BMI, diabetes, and hypercholesterolemia. Complementary functional genomic studies would shed more light on the underlying mechanism of human immunity associated with these genetic markers. The identified genetic factors may have the same role in susceptibility to similar respiratory infections with RNA viruses, like SARS, MERS and COVID-19. Future genetic association studies targeting these RNA viruses, especially COVID-19 is recommended. Studies on other ethnic groups would also shed light on possible ethnic variations in genetic susceptibility to respiratory RNA viruses.

*Trial registry* IR.PII.REC.1399.063

## Background

Influenza A virus is one of the most important risk factors for human and animal health leading to severe respiratory infections [1]. New strains cause the epidemics among humans and animals. These viruses use the host cell system for their replication and propagation and affect host cell signaling pathways [2]. While most of the antiviral studies have focused on the genetic factors of viral agents that determine the severity of influenza and their impact on host immunity, there is lack of a comprehensive understanding of the host genetics which determine the severity and susceptibility of the respiratory disease [3]. The family clustering of highly pathogenic influenza A /H5N1 cases has led to the hypothesis that host genetic plays a key and critical role in the susceptibility to influenza infection [4–6].

The severity of the disease is usually associated with the host factors such as age (over 65 years), pregnancy, obesity and underlying diseases including lung, cardiovascular, renal or hepatic disease, chronic metabolic disorders (including diabetes) or immunodeficiency diseases [7]. Underlying diseases such as diabetes and hypercholesterolemia are associated with the highest risk and the most common contributing factors [8–10]. Ethnicity has also been identified as another risk factor for the susceptibility to H1N1 pandemic in multiple populations in the North America and Australia [11]. A key point for the studies of host innate immunity factors is the observation of increased mortality among young adults and children without pre-identified risk factors [12].

It has been shown that host polymorphisms affect the development of certain diseases and disorders by coding altered genes products or altering the transcriptional settings. In order to understand the pathogenicity of influenza virus, several parameters of both host and virus must be considered. These factors mainly include virus pathogenicity, host acquired immunity and host hereditary factors that have been taken into consideration in determining the severity of the disease, particularly after the 2009 epidemic [3, 13].

Several potential genetic polymorphisms have recently been described for the susceptibility to influenza infection; including genes which encode complement decay-accelerating factor (CD55), complement 1 C1q binding protein (C1QBP), Fc fragment of IgG receptor IIa (FCGR2A), interferon induced transmembrane protein 3 (IFITM3), tumor necrosis factor (TNF), replication protein A interacting protein (RPAIN), lymphotoxin alpha (LTA), and killer-cell inhibitory receptor (KIR) [14–18].

Genetic polymorphisms vary among individuals in different populations and usually include single nucleotide polymorphisms (SNP).

For example, the **IFITM3** restricts the transcription of some viruses, especially those that enter the cell through endocytosis, such as influenza, HIV, and yellow fever [19–22]. In a study, the C allele frequency of IFITM3 rs12252 was shown high in Han Chinese population (MAF = 0.528) [23]. This means that IFITM3 gene affects the severity of the disease. The expression of the rare rs12252-C allele in the IFITM3 gene results in a truncated protein that lacks 21 amino acids at the end of the amine, which reduces its impact on influenza infection control [23].

In our previous study, we examined rs12252 in the IFITM3 gene in an Iranian population from the selective provinces [24]. That was the first study to evaluate the association of IFITM3 rs12252 polymorphism with susceptibility to mild influenza with regards to diabetes, hypercholesterolemia and BMI in an Iranian population. Our results showed a significant positive association between mild influenza and the heterozygote C allele at rs12252 (*P* = 0.008), not the homozygote C allele (*P* = 0.406). The carriers of the C allele (CT + CC genotype) were 5.92 times more at risk of mild influenza compared to the homozygote T allele (*P* = 0.007) [24].

Mannose-Binding Lectin-2 (**MBL-2**) gene is a member of the collagen-containing C-type lectin family. It is part of the innate immune system which is important for complement activation and opsonization [25]. MBL-2 encodes a calcium-dependent protein that plays an important role in innate immunity. The amount of circulating MBL-2 is mainly due to the single nucleotide polymorphisms occur in exon 1 and the promoter region. Different alleles of MBL-2 are associated with susceptibility to different infections [26–28].

Tumor necrosis factor- α (**TNF-α**) is a potent proinflammatory cytokine that plays role in the pathogenesis and development of various infectious diseases. However, **IL-10** is a potent anti-inflammatory cytokine that plays an important role in regulating and reducing the cytotoxic inflammatory responses. Several polymorphisms in the promoter regions of these genes have been shown that affect directly the gene transcription and are associated with the development and progression of autoimmune and infectious diseases [29, 30].

**CD55** or DAF (Decay-Accelerating Factor), encodes a glycoprotein involved in complement cascade regulation and is active in protecting host cells from damage of pathogenic microorganisms [31]. Studies have shown that the T/T genotype in the rs2564978 CD55 gene promoter is associated with severe influenza A (H1N1) pdm09 [32].

Two SNPs in the **C1QBP** (rs3786054) and **FCGR2A** (rs1801274) genes have been reported to be associated with the cases of severe influenza in Mexico in 2009 [17]. The C1QBP gene encodes a protein that binds to the spherical heads of C1q molecules and activates the classical complement pathway [17], while FCGR2A encodes a member of the Fc immunoglobulin receptor family which are found on the surface of phagocytic cells such as macrophages and neutrophils. It is involved in the process of phagocytosis and clearance of immune complexes [33].

**RPAIN** encodes a nucleoprotein that is involved in RNA movement and establishment [34]. The rs8070740 SNP in RPAIN has been shown to affect host immune response, or influenza A/H1N1 virus replication [17].

A significant association was also found between rs9856661 from an **unknown** gene located on chromosome 3 with the incidence of severe pneumonia in the patients with A/H1N1 virus infection [17].

Consequently, based on this background and our previous study findings, further studies are needed to confirm the earlier findings and to explore if further SNPs are associated with susceptibility to mild flu in the Iranian population. Identifying genetic markers associated with mild flu can give us hints and indications about the mechanisms of susceptibility and resistance to this virus. Identifying the pathogenic and resistance mechanisms may have applications in drug and vaccine design. Tracking the host genetic factors in susceptibility and resistance to influenza virus (as a model for the respiratory RNA viruses) may be the basis for understanding the pathogenicity of similar viruses such as coronavirus. Twelve SNPs in 9 genes located on 6 chromosomes with partial global minor allele frequency (GMAF) were selected and analyzed using iPLEX® and MassARRAY® system to genotype the desired alleles.

## Methods

### Ethics statement

The study objectives and processes were explained to all participants. Written informed consent was then obtained from them. The ethics committee of the Pasteur Institute of Iran reviewed and approved the protocol of this study (Ethics code: IR.PII.REC.1395.3).

### Participants and sample collection

Nasopharyngeal swab samples were collected as fluid specimens in Viral Transport Media (VTM). The samples were collected from 93 patients who were suspected to influenza A/H1N1 and referred to primary care centers of Markazi, Semnan, and Zanjan provinces (central Iran) from March 2015 to December 2018. Infection with influenza A/H1N1 was confirmed by real-time PCR test. Our exclusion criteria were respiratory comorbidities, immune suppressive drugs consumption, and previous vaccination against influenza virus [24]. Based on real-time PCR test results, patients were classified into influenza A/H1N1 positive (n = 49) and negative (n = 44) groups. The latter group was considered as having influenza-like illness (ILI).

Participants’ age (year), sex, weight (kg), and height (cm) were assessed at the time of their clinic visit. Diabetes (positive/negative) and hypercholesterolemia (positive/negative) status was also determined by the physician. Twelve SNPs in nine genes located on six chromosomes were genotyped. Genetic data of the SNPs analyzed are shown in Table 1.

**Table 1:** Genetic data of the SNPs analyzed

SNP	Chromosome	Gene	Location	Allele	GMAF
rs8070740	17	RPAIN	3ˈUTR	A > G	–
rs1801274	1	FCGR2A	Coding sequence	A > C / A > G	0.4302
rs1800451	10	MBL-2	Coding sequence	C > T	0.05466
rs2564978	1	CD55	Upstream transcript variant	T > C	0.2856
rs9856661	3	Unknown	–	A > C	–
rs361525	6	TNF-α	Intron variant	G > A	–
rs1800450	10	MBL-2	Missense variant	C > T	0.1212
rs3786054	17	C1QBP	Intron variant	G > A	–
rs1800871	1	IL-10	Intron variant	A > G	0.4086
rs1800872	1	IL-10	Intron variant	T > G	0.4091
rs1800896	1	IL-10	Intron variant	T > C	0.3026
rs1800629	6	TNF-α	Upstream transcript variant	G > A	0.0955

UTR: untranslated region; GMAF: Global minor allele frequency

### One-step quantitative real-time PCR for IAV detection

Viral RNA was extracted from fluid specimen using High Pure Viral RNA Kit, according to the manufacturer’s instructions (Roche, Switzerland). The extracted RNA was re-suspended in 50 μl Elution Buffer and stored at – 80 °C for real-time PCR assay. Primers and probes were designed and synthesized by SinaClon Co. (Iran) based on the latest WHO guideline. Real-time PCR reactions were performed as mentioned before [24].

### Genotyping of SNPs

#### Human DNA extraction

All genomic DNA samples were extracted from the specimens using DNP™ High yield DNA Purification Kit, according to the manufacturer’s instructions (Cinnagen, Iran) as stated before [24].

#### IPLEX® method and MassARRAY® system for genotyping the allelic variants

The extracted DNA samples were transferred to Inqaba Biotech Co, South Africa, to determine the genotypes of the allelic variants using iPLEX® and MassARRAY® platform (Sequenom MassARRAY® System, San Diego, USA). This SNP genotyping method involves an initial PCR reaction specific to the locus followed by single base extension using mass-modified primers containing terminal deoxyribonucleotides that binds immediately upstream of the polymorphism site. Using Matrix-Assisted Laser Desorption/Ionization-Time Of Flight (MALDI-TOF) Mass Spectrometry (MS), the specific mass of the amplified primer determines the SNP allele. This procedure involves the following steps: Pre-PCR for DNA (5 to 50 ng /µl) and oligonucleotides preparation, PCR for target locus amplification using designed primers, Post-PCR for purification of the reaction with SAP (Shrimp alkaline phosphatase), Single base amplification with iPLEX system, Cleaning products with resin, Deployment of amplified products on SpectroCHIPs, and Products tracking with Mass Spectrometry with appropriate MALDI-TOF matrix [35]. The specifications of the oligos for iPLEX SNP genotyping are shown in Table 2.

**Table 2:** The specifications of the oligos for iPLEX SNP genotyping

Name of the Oligo	Sequence of Oligo
rs8070740_W1_F	ACGTTGGATGGTCATAAGACCCAGATAGGC
rs8070740_W1_R	ACGTTGGATGCAGACAAACCTAATGCTGCC
rs8070740_W1_E	ATGCTGCCCTGCTA
rs1801274_W1_F	ACGTTGGATGCTTCCAGAATGGAAAATCCC
rs1801274_W1_R	ACGTTGGATGCTGTGACTGTGGTTTGCTTG
rs1801274_W1_E	AGGTGGGATCCAAA
rs1800451_W1_F	ACGTTGGATGAGAACAGCCCAACACGTACC
rs1800451_W1_R	ACGTTGGATGAGTGATTGCCTGTAGCTCTC
rs1800450_W1_E	agAAGATGGGCGTGATG
rs1800451_W1_F	ACGTTGGATGAGAACAGCCCAACACGTACC
rs1800451_W1_R	ACGTTGGATGAGTGATTGCCTGTAGCTCTC
rs1800451_W1_E	TGGTTCCCCCTTTTCT
rs2564978_W1_F	ACGTTGGATGGAACAATGTTCACTCCCTAC
rs2564978_W1_R	ACGTTGGATGCGTCATCTCCTAGAACACTC
rs2564978_W1_E	ACTCCCTACTGTGTTA
rs9856661_W1_F	ACGTTGGATGTGCCAGCTTAGAAAACCTCC
rs9856661_W1_R	ACGTTGGATGACCCACAGTGAACCTACAGA
rs9856661_W1_E	TGCATGGCAGAGGGCT
rs361525_W1_F	ACGTTGGATGGGATTTGGTGGGCAAAAGTC
rs361525_W1_R	ACGTTGGATGAGATGTCAAACAGGGACTGC
rs361525_W1_E	GGACTGCAAATTTCACA
rs3786054_W1_F	ACGTTGGATGTATGCAACTCTGGCAGGCAC
rs3786054_W1_R	ACGTTGGATGTCTCTTGACCTCGTGATCTG
rs3786054_W1_E	aggCCATTCCACACAAGA
rs1800871_W1_F	ACGTTGGATGATGCTAGTCAGGTAGTGCTC
rs1800871_W1_R	ACGTTGGATGGGTGTACCCTTGTACAGGTG
rs1800871_W1_E	CTTGTACAGGTGATGTAA
rs1800872_W1_F	ACGTTGGATGTCCTCAAAGTTCCCAAGCAG
rs1800872_W1_R	ACGTTGGATGAAAGGAGCCTGGAACACATC
rs1800872_W1_E	ccctACTGGCTTCCTACAG
rs1800896_W1_F	ACGTTGGATGATTCCATGGAGGCTGGATAG
rs1800896_W1_R	ACGTTGGATGGACAACACTACTAAGGCTTC
rs1800896_W1_E	tgACCTATCCCTACTTCCCC
rs1800629_W1_F	ACGTTGGATGGGAGGCAATAGGTTTTGAGG
rs1800629_W1_R	ACGTTGGATGCTGATTTGTGTGTAGGACCC
rs1800629_W1_E	cgGGAGGCTGAACCCCGTCC

#### Genetic and statistical analysis

Normality of continuous variables was confirmed by Kolmogorov Smirnoff test. Continuous and categorical data were described by mean ± SD, and number (percentage), respectively. Distribution of demographic and clinical characteristics was compared between influenza A/H1N1 group and the ILI group. Participants’ mean age and BMI was compared between both groups using Independent t-test. The two groups were compared regarding their gender, diabetes and hypercholesterolemia status using Pearson’s Chi square test.

Genotype frequency of each SNP was compared between influenza A/H1N1 group and the ILI group using Pearson’s Chi square test. Where expected genotype frequencies in more than 20% of the cells were less than five, Fisher’s exact test was used instead. Univariable logistic regression analysis was used to test the size of each genotype crude effect on influenza A/H1N1. Investigated demographic and clinical characteristics whose distribution differed significantly between the two groups were included into a multivariable logistic regression in order to test the adjusted effect of each allele on influenza A/H1N1.

All statistical tests were two-tailed with a type I error of 0.05. Statistical analyses were performed in Stata software (version 11; Stata Corp, College Station, TX, USA).

## Results

Demographical and clinical characteristics of influenza A/H1N1 patients and influenza-like illness (ILI) patients are shown in Table 3. In both the A/H1N1 and ILI groups, participants were equally of both genders (*P*_Pearson’s chi square_ = 0.460). The mean age of participants in both groups was 45 years. Participants in the ILI group were significantly of older ages than the A/H1N1 group (mean age: 56.5 vs. 46.2 years, respectively. *P*_t-test_ = 0.05). Mean BMI levels was higher in the A/H1N1 group (mean difference = 2.2 kg/m2). This association, however, was not statistically significant (*P*_t-test_ = 0.09). There was also a statistically significant association between diabetes (*P*_Pearson’s chi square_ = 0.001) and hypercholesterolemia (*P*_Fisher’s exact test_ = 0.03) statuses and A/H1N1, in a way that the frequency of these comorbidities was lower in the A/H1N1 group rather than the ILI group (Table 3).

**Table 3:** Demographical and clinical characteristics of influenza A/H1N1 patients and influenza-like illness (ILI) patients

Characteristics	Groups		
	Influenza A/H1N1	ILI	*P* value
*Sex*			
Male	26 (53.1)	20 (45.5)	0.463^§^
Female	23 (46.9)	24 (54.5)	
*Age*_(Year)_			
Mean ± SD	46.2 ± 22.5	56.5 ± 23.7	0.05*
< 45	21 (42.9)	10 (22.7)	**–**
45–64	7 (14.2)	12 (27.3)	
≥ 65	21 (42.9)	22 (50.0)	
BMI _Mean ± SD_	26.4 ± 1.1	24.2 ± 0.7	0.09*
*Diabetes*			
Negative	42 (85.7)	24 (54.5)	0.001^§^
Positive	7 (14.3)	20 (45.5)	
*Hypercholesterolemia*			
Negative	46 (93.9)	34 (77.3)	0.03^¤^
Positive	3 (6.1)	10 (22.7)	

*Independent t-test

^§^Pearson’s chi square test

^¤^Fisher’s exact test

Logistic regression analysis showed that possessing G/G genotype in the rs3786054 and rs8070740 loci significantly increases the risk of H1N1 influenza while compared to the A/A genotype in these loci (OR_rs3786054_ = 7.3; OR_rs8070740_ = 5.4). The direction of this association was the same for the G/A genotypes, although with smaller magnitude (OR_rs3786054_ = 2.6 and OR_rs8070740_ = 1.4); however, these associations were not statistically significant (Table 4). The results of the multivariable logistic regression analysis also showed that possessing the G allele in either rs3786054 or rs8070740 loci, significantly increases the risk of H1N1 influenza up to 3.3 folds, regardless of the patient’s age, BMI, diabetes, and hypercholesterolemia (Table 5).

**Table 4:** Frequency of 12 SNPs and their association with influenza A/H1N1 infection

Gene and genotype	Groups		P	Crude OR^**§**^	95% CI
	ILI n (%)	Influenza A/H1N1 n (%)			
rs1800450*					
CC	33 (84.6)	40 (88.9)	0.734	1	−
TT	1 (2.6)	0 (0)		−	−
TC	5 (12.8)	5 (11.1)		0.8	0.2, 3.1
rs1800451*					
CC	38 (97.4)	45 (100)	0.464	−	−
CT	1 (2.6)	0 (0)		−	−
rs1800629*					
GG	36 (85.7)	39 (84.8)	0.268	1	−
AA	2 (4.8)	0 (0)		−	−
GA	4 (9.5)	7 (15.2)		1.6	0.4, 6.0
*rs1800871*					
GG	17 (43.6)	19 (44.2)	0.425	1	-
AA	2 (5.1)	6 (14.0)		2.7	0.5, 15.1
GA	20 (51.3)	18 (41.8)		0.8	0.3, 2.0
*rs1800872*					
GG	18 (45.0)	18 (47.4)	0.616	1	−
TT	2 (5.0)	4 (10.5)		2.0	0.3, 12.3
GT	20 (50.0)	16 (42.1)		0.8	0.3, 2.0
*rs1800896*					
CC	2 (5.3)	4 (9.8)	0.832	1	−
TT	20 (52.6)	20 (48.8)		0.5	0.1, 3.1
CT	16 (42.1)	17 (41.5)		0.5	0.1, 3.3
*rs1801274*					
GG	5 (12.2)	9 (22.0)	0.056	1	−
AA	12 (29.3)	19 (46.3)		0.9	0.2, 3.3
GA	24 (58.5)	13 (31.7)		0.3	0.1, 1.1
*rs2564978*					
CC	25 (65.8)	29 (69.1)	0.938	1	−
TT	3 (7.9)	4 (9.5)		1.2	0.2, 5.6
CT	10 (26.3)	9 (21.4)		0.8	0.3, 2.2
*rs361525*					
GG	8 (20.5)	10 (23.8)	0.681	1	−
AA	14 (35.9)	11 (26.2)		0.6	0.2, 2.1
GA	17 (43.6)	21 (50.0)		1.0	0.3, 3.1
*rs3786054*					
AA	7 (23.3)	2 (5.9)	0.027	1	−
GG	11 (36.7)	23 (67.7)		**7.3**	**1.3, 41.1**
AG	12 (40.0)	9 (26.5)		2.6	0.4, 15.8
*rs8070740*					
AA	13 (31.7)	28 (65.1)	0.007	1	−
GG	10 (24.4)	4 (9.3)		**5.4**	**1.4, 20.4**
GA	18 (43.9)	11 (25.6)		1.5	0.4, 6.1
*rs9856661*					
CC	5 (12.5)	4 (9.8)	0.272	1	−
AA	19 (47.5)	27 (65.8)		1.8	0.4, 7.5
CA	16 (40.0)	10 (24.4)		0.8	0.2, 3.6

*Odds ratio has not been estimated for the genotypes with zero observations

^§^Significant odds ratios (ie., *P* < 0.05) are bolded (*P*rs 3786054: 0.0140; *P*rs 8070740: 0.0093). ILI: influenza like illness; OR: odds ratio

**Table 5:** Crude and adjusted allelic risk of influenza A/H1N1 infection in relation to alleles of polymorphisms studied

Gene and genotype*	Crude OR^¤^	95% CI	Adjusted OR^§¤^	95% CI
*rs1800450* (/C)				
T	0.7	0.2, 2.5	0.9	0.1, 6.0
*rs1800629* (/G)				
A	1.1	0.3, 3.5	1.1	0.1, 8.5
*rs1800871* (/A)				
G	1.0	0.4, 2.5	1.1	0.3, 4.1
*rs1800872* (/G)				
T	0.9	0.4, 2.2	1.0	0.3, 3.7
*rs1800896* (/T)				
C	1.2	0.5, 2.8	2.5	0.6, 9.4
*rs1801274* (/G)				
A	2.1	0.8, 5.2	1.5	0.4, 6.7
*rs2564978* (/C)				
T	0.9	0.3, 2.2	0.4	0.1, 1.6
*rs361525* (/A)				
G	1.6	0.6, 4.1	1.9	0.4, 8.6
*rs3786054* (/A)				
G	**3.3**	**1.3, 11.1**	**3.3**	**1.1, 22.0**
*rs8070740* (/A)				
G	**3.3**	**1.7, 10**	**3.3**	**1.1, 14.3**
*rs9856661* (/A)				
C	**2.0**	**1.1, 5.0**	**2.0**	**1.0, 10.0**

*Allele frequencies have been considered for defining the baseline category in the logistic regression analysis. In fact, the alleles with higher frequency were considered as the baseline category in the logistic regression

^¤^Significant odds ratios (ie., *P* < 0.05) are bolded

^§^The odds ratios are adjusted for the confounding effect of age, body mass index, diabetes (positive/negative), and hypercholesterolemia (positive/negative). OR: odds ratio

The crude effect of rs9856661 genotypes on the H1N1 influenza risk was not statistically significant, probably due to small number of observations within each genotype category. The allelic OR, however, showed that possessing the A allele (rather than the C allele) significantly increased the risk of H1N1 influenza up to two folds both in the crude analysis and after adjustment for the abovementioned demographics and comorbidities (Table 5). The association of other SNPs with H1N1 influenza was not statistically significant either in crude or adjusted analyses (Tables 4 & 5).

## Discussion

In the present study, we investigated the genetic susceptibility of the host to influenza virus infection by incorporating hypercholesterolemia and diabetes factors. The alleles and their genotypes were examined to determine whether the polymorphisms of the studied genes are associated with the susceptibility to influenza virus in this population with a preference for hypercholesterolemia and diabetes. Host and pathogen genetic determinants are important to evaluate pathogenesis of infectious disease. Several polymorphisms which have been known for the susceptibility to influenza infection include CD55, C1QBP, FCGR2A, IFITM3, TNF, RPAIN, LTA and KIR [14–18]. The protein products of the tested SNPs were reported to be associated with inflammatory reactions [36].

In this study, we examined a wide range of human genetic variants in different genes involved in immune response against viral infection in order to explore their potential association with mild influenza in human. Mild influenza causes mild respiratory infection, pulmonary dysfunction and clinical manifestation. It can resolve on its own without significant symptoms and without need for hospitalization. From 12 studied SNPs that are believed to be associated with immune response against viral infection, nine (rs1800450, rs1800629, rs1800871, rs1800872, rs1800896, rs1801274, rs1801274, rs2564978, rs361525) did not show any significant association with mild flu in our study. Three SNPs (rs3786054, rs8070740, rs9856661), however, revealed evidence on such associations. Possessing G allele (vs. A) in rs3786054 and rs8070740 loci is associated with a threefold increase in the chance of mild flu when compared to ILI patients. Possessing C allele (vs. A) in the rs9856661 locus also increases the chance of mild flu up to 2 folds.

In regards to **IFITM3**, Zhang et al*.,* estimated that patients carrying the CC genotype in their rs12252 IFITM3 were six times more at risk of severe infection than those with the CT and TT genotypes. Higher levels of the CC allele in the Han Chinese population than in White population may put the Chinese at higher risk for developing severe illness following influenza virus infection. IFITM3 may also play an important role in virus replication and proliferation after initial infection [23]. Recently, with extensive analysis, the close association of the rs12252-C allele with influenza susceptibility has been demonstrated in two Han Chinese and English whites populations [37]. However, in some populations such as Caucasian (MAF = 0.041) [23] and infants and children populations [38], little correlation was found between this allele and susceptibility to influenza. Zhang et al*.,* in 2013 demonstrated the association of the C allele at rs12252 in the IFITM3 gene in different populations of Chinese, Japanese, Northern European and English with the 2009 pandemic influenza [23]. In a recent review study, the effect of rs12252 in IFITM3 gene on influenza infection was investigated demographically [39]. In our previous study on IFITM3, BMI, diabetes, and hypercholesterolemia in an Iranian population, no association was found between mild influenza and the homozygote allele rs12252 C and these underlying factors. The carriers of the C allele were more at risk of mild influenza compared to the homozygote T allele [24].

**TNF-α** functions in homeostasis and disease pathogenesis. It induces pathogenic epigenetic modifications and promotes enhanced inflammatory responses even to minor environmental challenges [40]. **IL-10** plays pleiotropic role in immunoregulation and inflammation. It can block NF-kB activity and is involved in the regulation of the JAK-STAT signaling pathway. Mutations in this gene have been shown to be associated with the increased susceptibility to HIV-1 infection [provided by RefSeq, May 2020]. This cytokine selectively impacts the expression of Th17-associated cytokines during influenza infection [41]. IL-10 can impair T cell priming in the early stages of adaptive immunity, a mechanism that viruses use to promote their persistence by infecting antigen presenting cells (APCs). IL-10 effects are more subtle in the immune response during acute infections [42].

The **TNF** polymorphisms were shown to be associated with risk of infection by influenza A/H1N1 virus during the pandemic in Mexico in 2009. Carriers of the rs1800629 G/A genotype were associated with high levels of blood urea nitrogen [18]. The role of rs361525 of TNF-α gene located on the short arm of chromosome 6 in the course of infection with 2009 H1N1 influenza virus was confirmed [14]. TNF-α rs361525 G/A genotype was associated with an increased risk of disease severity. TNF-α rs1800629 G/G genotype and **IL-10** rs1800872 and rs1800896 were also associated with increased disease severity [43].

Genotype G/G and G/T of IL-10 rs1800872 were associated with increased risk of infection with influenza A/H3N2 virus. This might be due to anti-inflammatory nature of IL-10 that prevents NK and T cell activities to affect the strong inflammatory action after first infection [44]. In the study by Keshavarz et al*.,* the frequencies of IL-10 rs1800872 and IL-28 rs8099917 were shown not to be associated with influenza disease [45] which was in accordance with our results.

The IL-10 rs1800872 is related to increased intensity of autoimmune and infectious diseases which can control the transcription and expression of IL-10 [46]. The same polymorphism was investigated by Ferdinand et al*,.* They studied the frequency of TNF and MBL SNPs, located on the chromosomes 6 and 10, respectively, in children and adolescents with fatal influenza infection and compared the outcome with the control group. They reported that influenza cases leading to death with concomitant methicillin-resistant Staphylococcus aureus (MRSA) infection had a higher incidence of low-production MBL2 genotype compared to the cases of death without MRSA co-infection [47]. A study conducted on 246 patients infected with A/H1N1pdm09 virus in India, suggested that SNPs in the IL-10 and TNF-α genes might be associated with disease severity in influenza A/H1N1pdm09-infected patients. In this study, TNF-α rs1800629, IL-10 rs1800872, IL-10 rs1800896 were genotyped by PCR along with other genotypes. TNF-α rs1800629 G/A genotype showed significantly higher frequency in severe cases compared to the mild cases. IL-10 rs1800896 G allele, in dominant mode, was significantly negatively associated with the disease severity. IL-10 rs1800896 C/A genotype was significantly associated with fatality in influenza A/H1N1pdm09 infections [48], however, in our study we could not find any association of these SNPS with influenza infection.

**MBL-2** gene different alleles are associated with susceptibility to different infections [26–28].

This gene encodes the soluble mannose-binding lectin which belongs to the collectin family and is considered as an important element in the innate immune system. This protein binds to mannose and N-acetylglucosamine on different microorganisms and viruses including influenza virus, HIV and SARS-CoV. This binding activates the classical complement pathway [provided by RefSeq, Jun 2020].

Codon 54 of this gene is more prevalent in White population than codons 52 and 57. Individuals with A allele in codon 54 have lower levels of MBL-2 protein compared to those carrying codon 52. This MBL-2 protein is disabled for classical complement activation [49]. Among the polymorphisms of this gene, codon 54 is associated with susceptibility to infection [50, 51]. Recently, in the evaluation of MBL gene variants in pediatric influenza-related illness, MBL deficiency was suggested as no serious risk factor for very severe influenza infection in children and adolescents [52].

In a matched case–control study conducted in Brazil, the association between MBL2, CLEC5A, ITGB3 and CCR5 genes and dengue severity was investigated in children. They found no association in single SNP analysis. However, the allele rs7095891G/rs1800450C/ rs1800451C/ rs4935047A/ rs930509G/ rs2120131G/ rs2099902C were significantly associated to the risk of severe dengue when MBL2 SNPs were combined in haplotypes [53], however, in our study we could not find any association of rs1800450 and rs1800451 SNPs with influenza infection.

**FCGR** is a glycoprotein which binds to the Fc region of IgG and activates immune response to control pathogens [54]. FCGR2A is one of the five genes located on chromosome 1, which encode receptors with low affinity for this glycoprotein. The activation or inhibition of these receptors regulates the local inflammatory response [55]. This gene encodes a cell surface receptor found on phagocytic cells such as macrophages and neutrophils, and is involved in the process of phagocytosis and clearing of immune complexes [provided by RefSeq, Oct 2008].

The genetic variant of the IgG Fc receptor at position 131 has been shown to be associated with susceptibility to inflammatory and infectious diseases like Kawasaki disease [56] and pneumococcal diseases [57]. Researchers studied the rs1801274 variant of the FCGR2A gene and concluded that this variant may be related to greater susceptibility to severe forms of flu [17]. Maestri and colleagues investigated this SNP for His131Arg substitution in Brazilian admixed population diagnosed with H1N1 pdm09 infection. They detected no association of rs1801274 polymorphism with severe disease in this population [15]. In the study on the patients from northern Greece suffered from influenza A(H1N1)pdm09 disease, no significant difference was either detected for FCGR2A genotypes neither with fatality nor disease severity [58] which was in line with our study outcome. However, this study found relationship between rare T/T genotype of **CD55** rs2564978 and rare A/A genotype of **C1QBP** rs3786054 with increased death risk but not with disease severity [58].

CD55 may play an important role in the presentation of disease. It functions to protect the host cells from complement attack and inhibits the complement cascade. The high expression level of CD55 in human lung may reflect its critical role to prevent lung from complement-mediated tissue damage as a result of long-term evolutionary pressure [16].

C1QBP inhibits the complement activation [59]. It was shown to be involved in HIV-1 replication, probably by contributing to splicing of viral RNA [60].

Carriers of T/T rs2564978 genotype in CD55 gene in Chinese population have been shown to be significantly associated with severe H1N1 infection [16]. Studies have shown that patients with T/T genotype at rs2564978 in **CD55** gene contained lower levels of CD55 in peripheral blood mononuclear cells compared to the patients with the C/C and C/T genotypes which cannot regulate complement cascade [32]. The association between IFITM3 rs12252, and CD55 rs2564978 SNPs and influenza H7N9/H1N1 pdm09 clinical outcomes was examined in Chinese population. The over-expression of homozygote CC genotype at rs12252 in IFITM3 among fatal cases was detected and CD55 TT genotype at rs2564978 was linked to severity of the disease [61]. The CD55 rs2564978 SNP did not show significant association with influenza in our study but C1QBP rs3786054 G allele (vs. A) showed association with a threefold increase in the chance of mild flu.

In regards to rs8070740 and rs9856661 at **RPAIN** and **unknown genes**, respectively, it was suggested that these genes polymorphisms might impact the susceptibility to severe pneumonia in A/H1N1 infection [17].

RPAIN in short name; hRIP, mediates the import of replication protein A (RPA) complex into the nucleus, possibly via interaction with importin beta. The isoform 2 of this gene is sumoylated by ubiquitin-like protein and mediates the localization of RPA complex into the nucleus, thereby participating in DNA metabolism [62].

Our study confirmed these results as rs9856661C allele increased the chance of mild flu up to 2 folds in Iranian population.

Our study has notable strengths, including: a) novel method of genotyping; b) study on the Iranian ethnicity for these SNPs for the first time; and c) high number of SNPs analyzed in a single study. In spite of the value of the first genetic association study of its kind in Iran, this study has a number of limitations, particularly its small sample size. Further studies on larger sample sizes will be warranted.

## Conclusion

The results showed that possessing the G allele in either rs3786054 or rs8070740 loci in C1QBP and RPAIN genes, respectively, increased the risk of H1N1 infection up to 3.3 folds, regardless of the patient’s age, BMI, diabetes, and hypercholesterolemia. Future functional genomic studies on identified genetic markers may provide clues to the molecular targets that can be used for therapies, as well as effective vaccines. Given multiple factors such as vaccination, virus subtype and host genetic factor play role in the immune response against influenza, systems biology methods are needed to illuminate the interaction between these factors and their role in susceptibility to influenza. The identified genetic and clinical factors may also be associated with susceptibility to other respiratory RNA viruses, like Severe Acute Respiratory Syndrome (SARS), Middle East Respiratory Syndrome (MERS) and COVID-19. Future genetic association studies targeting these RNA viruses, especially COVID-19 is recommended to provide better understanding of host the immune response against the virus [63, 64]. A better understanding of the biological pathways leading to severe viral disease can be helpful for more effective treatments. New discoveries may generate hypotheses for drug repurposing and may estimate the country's need for vaccine design and production. Also, studies on other ethnic groups would also shed the light on possible ethnic variations in genetic susceptibility to respiratory RNA viruses.

## Data Availability

All data in case of need are available.

## Abbreviations

APCs: Antigen presenting cells
C1QBP: Complement 1 C1q binding protein
CD55: Complement decay-accelerating factor
DAF: Decay-accelerating factor
FCGR2A: Fc fragment of IgG receptor IIa
GMAF: Global minor allele frequency
IAV: Influenza A virus
IFITM3: Interferon induced transmembrane protein 3
ILI: Influenza like illness
KIR: Killer-cell inhibitory receptor
LTA: Lymphotoxin alpha
MALDI-TOF: Matrix-assisted laser desorption/ionization-time of flight
MBL-2: Mannose-binding Lectin-2
MS: Mass spectrometry
OR: Odds ratio
RPA: Replication protein A
RPAIN: Replication protein A interacting protein
SAP: Shrimp alkaline phosphatase
SNP: Single nucleotide polymorphism
TNF: Tumor necrosis factor
UTR: Untranslated region
VTM: Viral transport media
